# Dr. Klein on Antitoxin

**Published:** 1895-03-23

**Authors:** 


### Dr. Klein on Antitoxin.

The March issue of *Science Progress* contains a very interesting article on "Antitoxin" from the pen of Dr. Klein, who writes with special authority upon the subject which at present occupies so prominent a place in medical thought. The article is brief, unencumbered with detail, and places the matter in a very clear light. Starting with the long-observed facts of immunity from certain infective diseases, both natural and acquired, it describes the various stages of discovery and phases of opinion which have led up to our present position. Dr. Klein shows that immunity is not acquired by any process of exhaustion of any ingredient necessary to the life of specific disease-germs, and that infective diseases are not comparable in this point to fermentative processes, which come to an end when the particular substance on which the fermentation microbes live is exhausted. The next great step in our knowledge was the discovery of the toxins generated by specific microbes, and that these toxins are the active causes of the diseases produced by the presence and growth of the organisms. These toxins have not only been separated from cultures of micro-organisms grown in laboratories, and proved to be potent by the effects of injecting them into the blood and tissues of animals, but the same toxins have been separated from the blood of animals suffering from these infective diseases, and they have also been shown to lead to the production of other secondary chemical products—albumoses and alkaloids—by their action on the living tissues. The next link in the chain was forged when it was shown that immunity could be produced by repeated inoculations of a susceptible animal with non-fatal doses of toxin in gradually increasing quantity, and that this rendered the animal immune not only against a fatal dose of toxin but against inoculation with the bacilli themselves. This antitoxic power has been shown to reside in the blood of the animal and to be due to certain chemical substances in it rather than to any power or change in the cells of the blood or other tissues of the body. Having reached this point the inquiry was naturally directed to ascertain how this antitoxin is produced, and Dr. Klein considers that no definite answer to this question can be given. Some observers think that the toxin is actually converted into antitoxin by the action upon it of the living cells of the animal. Others, and to this view Dr. Klein evidently inclines, believe that the antitoxin is a direct product of the living cells produced under the stimulus or irritation of the toxin. There is an important practical difference between these two views. If antitoxin is altered toxin we may hope to prepare it artificially in our laboratories without the active agency of living animals. But if it is the product of some change in the cells of the organism, a change brought about by the influence on the cells of the toxin, we shall always have to seek for this agent in the blood of living animals, and shall not be able to prepare it artificially. But Dr. Klein adds as a last important fact that the substance of bacteria is itself antitoxic, and that immunity against infective disease is acquired not only from the antitoxin evolved from the cells of the diseased animal under the stimulus of the toxin, but from the débris of the specific organisms themselves. The therapeutical value of all this knowledge was suggested when it was shown that the antitoxin in the blood of one animal could be introduced into another animal and endow the latter with the immunity acquired by the former. No more striking therapeutical suggestion has ever been made, and we can only hope its success will be as startling.

